# Tracking antimicrobial stewardship activities beyond days of therapy (DOT): Comparison of days of antibiotic spectrum coverage (DASC) and DOT at a single center

**DOI:** 10.1017/ice.2022.312

**Published:** 2023-06

**Authors:** Hiroyuki Suzuki, Brett H. Heintz, Daniel J. Livorsi, Eli N. Perencevich, Michihiko Goto

**Affiliations:** 1 Center for Access & Delivery Research & Evaluation (CADRE), Iowa City Veterans’ Affairs Health Care System, Iowa City, Iowa; 2 Iowa City Veterans’ Affairs Health Care System, Iowa City, Iowa; 3 Department of Internal Medicine, University of Iowa Carver College of Medicine, Iowa City, Iowa

## Abstract

Even though antimicrobial days of therapy did not significantly decrease during a period of robust stewardship activities at our center, we detected a significant downward trend in antimicrobial spectrum, as measured by days of antibiotic spectrum coverage (DASC). The DASC metric may help more broadly monitor the effect of stewardship activities.

In the United States, days of therapy (DOT) is the preferred metric for measuring inpatient antimicrobial consumption.^1^ In addition, DOT is used for the National Healthcare Safety Network (NHSN) standardized antimicrobial administration ratio (SAAR), which is a comparison of observed DOT to predicted DOT.^2^ Although it is widely used, a major disadvantage of DOT is that it does not account for antimicrobial spectrum.

Antimicrobial stewardship programs (ASPs) promote optimal antimicrobial prescribing, including the avoidance of unnecessarily broad-spectrum antimicrobials, through a variety of strategies, such as clinical pathways, education, antimicrobial allergy assessments, and prospective audit and feedback (PAF).^3–5^ By avoiding unnecessarily broad-spectrum antimicrobials, ASPs can help decrease the risk of developing antimicrobial resistance and *Clostridioides difficile* infection without a negative impact on clinical outcomes.^6–8^

However, stewardship efforts to avoid broad-spectrum antimicrobials are not captured by DOT. DOT counts the number of unique antimicrobials per day without taking the spectrum of antimicrobials into account. Furthermore, if a patient is prescribed a combination of 2 or more types of antimicrobials, DOT could be higher while the antimicrobial spectrum is narrower (eg, piperacillin-tazobactam vs ceftriaxone and metronidazole). Although the NHSN provides several metrics that aggregate the use of antimicrobials with similar spectrums of activity (eg, narrow-spectrum β-lactam agents), no single summative metric in the NHSN captures the antimicrobial spectrum for all prescribed agents.

To overcome the limitation of existing metrics, we proposed a new metric for antimicrobial consumption: days of antibiotic spectrum coverage (DASC). DASC is calculated by multiplying DOT and the antibiotic spectrum coverage (ASC) score, which is a summative score of antimicrobial spectrum (Table 1).^9^ It is possible that DASC, which captures both antimicrobial spectrum and volume, could better reflect the effect of ASP activities than DOT, but no study has compared the 2 metrics over time in a facility with an active ASP. This study was designed to help address this knowledge gap.

**Table 1.** Antibiotic Spectrum Coverage (ASC) Scores for 77 Antibiotics

ASC Score	Antibiotic
1	None
2	Dicloxacillin, nafcillin, oxacillin, metronidazole, tinidazole
3	Penicillin G, penicillin V, cefadroxil, cefazolin, cefixime, cephalexin, erythromycin
4	Aztreonam, cefdinir, cefpodoxime, cefprozil, cefuroxime
5	Quinupristin-dalfopristin, rifampin, amoxicillin, ampicillin, cefiderocol, clarithromycin, dalbavancin, telavancin, vancomycin
6	Polymyxin B, colistin, linezolid, tedizolid, azithromycin, daptomycin, clindamycin, oritavancin, cefotetan, cefoxitin, ceftazidime, ceftriaxone, cefotaxime
7	Tetracycline, doxycycline, lefamulin, piperacillin, nitrofurantoin, sulfamethoxazole-trimethoprim, ceftaroline, amoxicillin-clavulanate
8	Minocycline, fosfomycin, cefepime, ceftolozane-tazobactam, ceftazidime-avibactam, ampicillin-sulbactam, amikacin
9	Chloramphenicol, ofloxacin, norfloxacin, ciprofloxacin, ertapenem, tobramycin, plazomicin, gentamicin
10	Ticarcillin-clavulanate
11	Gemifloxacin, piperacillin-tazobactam
12	Doripenem, imipenem-cilastatin, meropenem, levofloxacin
13	Imipenem-cilastatin-relebactam, moxifloxacin, meropenem-vaborbactam
14	Delafloxacin
15	Eravacycline, omadacycline, tigecycline
16	None

In this study, we evaluated the performance of DASC versus DOT within the Iowa City Veterans’ Affairs Healthcare System (ICVAHCS).

## Methods

This retrospective study was conducted at the ICVAHCS’s acute-care hospital, which has 83 inpatient beds including 10 ICU beds. The ICVAHCS provides care for veterans in eastern Iowa, western Illinois, and northern Missouri. We retrospectively analyzed inpatient antimicrobial use during calendar years 2017 through 2021. During the observational period, the ICVAHCS had an ASP led by 1 infectious diseases (ID) pharmacist (1.0 full-time equivalents or FTE) and 1–2 ID physicians (total, 0.5 FTE). The ASP monitored all inpatients on antimicrobials and performed prospective audit-and-feedback (PAF) every weekday. Our PAF process led to a mean of 9.7 (SD, ±1.9) documented real-time recommendations per week during the study period. Additional ASP activities included a monthly educational lecture to medical residents or students; promotion of a local antimicrobial-prescribing guide available via a free smartphone software application (cf, app); and routine assignment of a clinical pharmacist to each inpatient physician team for assistance with ordering antimicrobials.

Antimicrobial DOT data for each antimicrobial type were obtained from the Veterans’ Integrated Service Network (VISN) 23 ASP dashboard, which utilizes real-time antimicrobial usage data from the VHA Corporate Data Warehouse (CDW). We aggregated DOT based on published methodology.^10^ DASC was calculated based on the methodology described in Supplementary Table 1 (online). We did not calculate DASC for antimicrobials other than antibiotics because ASC was not available for antifungals or antivirals. Patient days (PD) was used as a denominator for both DOT and DASC.

Trends of DOT, DASC and DASC/DOT were analyzed using one-way linear regression accounting for the month of the year to address the variation in antimicrobial prescribing according to seasonality. All analyses were conducted using R version 3.5.0 software (R Foundation for Statistical Computing, Vienna, Austria).

Antimicrobial consumption data were readily available as a part of the operational quality improvement project. This study was reviewed by the University of Iowa and ICVAHCS Institutional Review Board and was determined to be a quality improvement initiative.

## Results

**Fig. 1.**
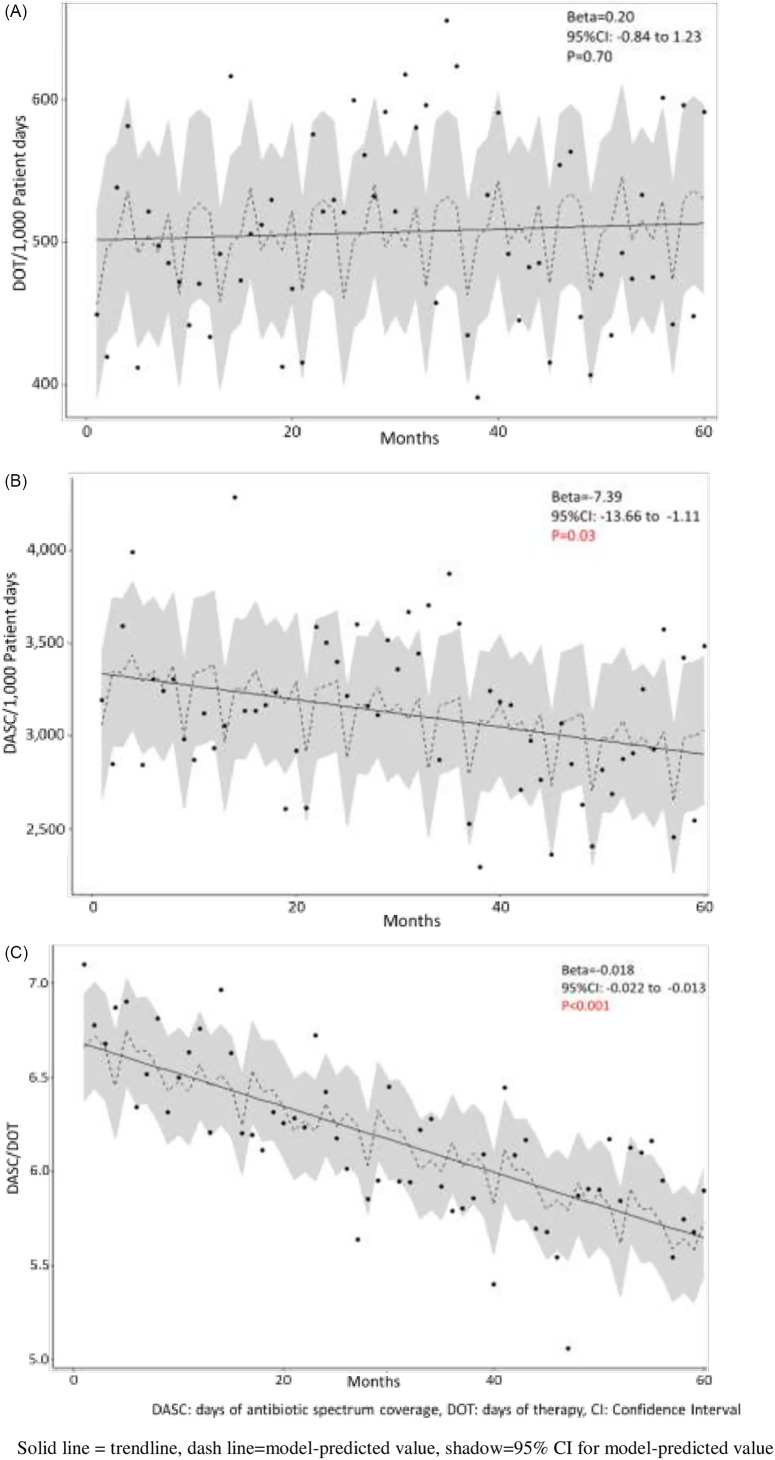
Antimicrobial consumption at the Iowa City VA Healthcare System during 2017–2021, using (A) days of therapy (DOT), (B) days of antibiotic spectrum coverage (DASC), and (C) DASC/DOT.

Monthly DOT per 1,000 PD at ICVAHCS are shown in Figure 1A. There was no significant increase or decrease in trend (β = 0.20; 95% CI, −0.84 to 1.23; *P* = .70). During the study period, DOT for antipseudomonal agents (piperacillin-tazobactam, cefepime or meropenem) decreased by 12% (87.9 DOT per 1,000 PD in 2017 to 77.2 DOT per 1,000 PD in 2021). Also, DOT for fluoroquinolones decreased by 54% from 38.5 to 17.7 DOT per 1,000 PD. And DOT for anti-methicillin-resistant *Staphylococcus aureus* agents (ie, vancomycin, daptomycin, or linezolid) decreased by 19% from 65.7 to 53.2 DOT per 1,000 PD. In contrast, DOT for ceftriaxone increased by 37% during the study period from 45.3 DOT to 62.0 DOT per 1,000 PD, and DOT for ampicillin-sulbactam or amoxicillin-clavulanate increased by 12% from 34.5 to 38.6 DOT per 1,000 PD. Monthly DASC per 1,000 PD are shown in Figure 1B. We detected a significant downward trend in monthly DASC (β = −7.39; 95% CI, −13.66 to −1.11; *P* = .03). Finally, Figure 1C describes monthly DASC/DOT. Monthly DASC/DOT also significantly decreased over the study period (β = −0.018; 95% CI, −0.022 to −0.013; *P* < .001).

## Discussion

In this study, our analysis revealed significant declines over 5 years in antimicrobial spectrum, as measured by DASC, while standard DOT remained stable. This decline in antimicrobial spectrum occurred in the context of robust ASP activities, which included guidance on the initiation of narrow-spectrum antimicrobials and on antimicrobial de-escalation.

Our findings suggest that DASC may be more responsive to stewardship activities than total DOT. Although the benefits of ASP activities at our center could be captured with antimicrobial-specific DOT for high-risk antimicrobials, such as carbapenems or fluoroquinolones, total DOT would be a less informative metric. This is because various stewardship processes at our hospital that encouraged the use of narrow-spectrum over broad-spectrum agents would not affect total DOT or, paradoxically, would increase total DOT if a combination of antimicrobials were prescribed. In contrast, ASP activity at our center did affect DASC, which incorporates both consumption and the spectrum of antimicrobials. Antimicrobial spectrum could be captured by tracking different SAAR antimicrobial agent categories, but DASC provides a summative metric that captures the spectrum of all antimicrobials and is therefore easier to use. Validation of DASC in different hospital settings is necessary before the metric is widely adopted.

This study had several limitations. First, we could not assess the exact reasons why DASC declined without patient-level data and a detailed assessment of ASP activities in this study. We believe ASP’s effort to avoid broad-spectrum antimicrobials was the main driver for the decline in DASC because the use of broad-spectrum antimicrobials, such as fluoroquinolones or antipseudomonal agents, declined while the use of narrower antimicrobials increased over the study period. Second, we did not assess how these new metrics were associated with antimicrobial-related clinical outcomes such as emergence of resistant organisms or *C. difficile* infection incidence. Possibly, reducing DASC was associated with less emergence of antimicrobial resistance, but this hypothesis needs to be confirmed by further research.

In conclusion, DASC per 1,000 patient days and DASC/DOT demonstrated the downward trend of antimicrobial spectrum at our center, which was not captured by DOT. We believe that DASC is a simple but useful metric that should be considered for wider adoption if future studies confirm its responsiveness to stewardship processes and its association with relevant clinical outcomes.

## Supplementary material

For supplementary material accompanying this paper visit https://doi.org/10.1017/ice.2022.312. click here to view supplementary material

## References

[1] Barlam TF , Cosgrove SE , Abbo LM , et al. Implementing an antibiotic stewardship program: guidelines by the Infectious Diseases Society of America and the Society for Healthcare Epidemiology of America. Clin Infect Dis 2016;62:e51–e77. 27080992 10.1093/cid/ciw118 PMC5006285

[2] The NHSN standardized antimicrobial administration ratio (SAAR). Centers for Disease Control and Prevention website. https://www.cdc.gov/nhsn/pdfs/ps-analysis-resources/aur/au-saar-guide-508.pdf. Accessed March 16, 2022.

[3] The core elements of hospital antibiotic stewardship programs. Centers for Disease Control and Prevention website. https://www.cdc.gov/antibiotic-use/healthcare/pdfs/hospital-core-elements-H.pdf. Published 2019. Accessed August 3, 2021.

[4] Trubiano JA , Thursky KA , Stewardson AJ , et al. Impact of an integrated antibiotic allergy testing program on antimicrobial stewardship: a multicenter evaluation. Clin Infect Dis 2017;65:166–174. 28520865 10.1093/cid/cix244 PMC5849110

[5] Chua KYL , Vogrin S , Bury S , et al. The penicillin allergy delabeling program: a multicenter whole-of-hospital health services intervention and comparative effectiveness study. Clin Infect Dis 2021;73:487–496. 32756983 10.1093/cid/ciaa653 PMC8326579

[6] Elligsen M , Walker SA , Pinto R , et al. Audit and feedback to reduce broad-spectrum antibiotic use among intensive care unit patients: a controlled interrupted time series analysis. Infect Control Hosp Epidemiol 2012;33:354–361. 22418630 10.1086/664757

[7] Fowler S , Webber A , Cooper BS , et al. Successful use of feedback to improve antibiotic prescribing and reduce *Clostridium difficile* infection: a controlled interrupted time series. J Antimicrob Chemother 2007;59:990–995. 17387117 10.1093/jac/dkm014

[8] Aldeyab MA , Kearney MP , Scott MG , et al. An evaluation of the impact of antibiotic stewardship on reducing the use of high-risk antibiotics and its effect on the incidence of *Clostridium difficile* infection in hospital settings. J Antimicrob Chemother 2012;67:2988–2996. 22899806 10.1093/jac/dks330

[9] Kakiuchi S , Livorsi DJ , Perencevich EN , et al. Days of antibiotic spectrum coverage (DASC): a novel metric for inpatient antibiotic consumption. Clin Infect Dis 2022;75:567–574. 34910130 10.1093/cid/ciab1034

[10] Antimicrobial use and resistance (AUR) module. Centers for Disease Control and Prevention website. https://www.cdc.gov/nhsn/pdfs/pscmanual/11pscaurcurrent.pdf. Accessed September 6, 2022.

