# Supplementary material for: Tracking antimicrobial stewardship activities beyond days of therapy (DOT): Comparison of days of antibiotic spectrum coverage (DASC) and DOT at a single center
Source: Infect Control Hosp Epidemiol. 2023 Jan 10;44(6):934–7. doi: 10.1017/ice.2022.312 (PMC10262162; doi:10.1017/ice.2022.312)
Supplement: Supplementary file 1 [file S0899823X22003129sup001.docx]

Supplemental table: A brief explanation of how days of antibiotic spectrum coverage (DASC) was calculated

The antibiotic spectrum coverage (ASC) score for each antibiotic was developed as a summative score of antibiotic activity against 11 wild-type microorganisms (methicillin-susceptible *Staphylococcus aureus*, *Streptococcus* species, *Enterococcus faecalis*, oral anaerobes, *Bacteroides fragilis*, *Moraxella catarrhalis*/*Hemophilus influenzae*, *Escherichia coli*/*Klebsiella pneumoniae*, *Enterobacter*/*Serratia*/*Citrobacter* spp., *Pseudomonas aeruginosa*, *Acinetobacter baumannii*, and atypical organisms (*Rickettsia*/*Chlamydophila*/*Mycoplasma*/*Legionella* spp.)) and 5 acquired resistance mechanisms (extended-spectrum β-lactamase–producing Enterobacteriales, methicillin-resistant *S. aureus*, penicillin-resistant *Streptococcus pneumoniae*, vancomycin-resistant *Enterococcus* spp., and carbapenem-resistant Enterobacteriales). ASC score was assigned through literature searches and if no explicit statement was found in the existing literature or there was a discrepancy between in vitro activity and clinical recommendations, we convened an expert panel, including infectious diseases specialists, microbiologists, and pharmacists, to form a consensus. DASC for each antibiotic was calculated by multiplying ASC for each antibiotic by Days of Therapy (DOT). Then DASC for each antibiotic was aggregated to calculate total monthly DASC. Finally, DASC/DOT was calculated by dividing total monthly DASC by total monthly DOT (DASC/DOT) to evaluate mean ASC for the month.

Example of ASC score

Cefazolin=3 (MSSA, *Streptococcus* spp., *E. coli*/*K.pneumoniae*)

Ceftriaxone=6 (MSSA, *Streptococcus* spp., *Moraxella*/*H. influenzae*, *E. coli*/*K.pneumoniae*, *Enterobacter*/*Serratia*/*Citrobacter*, PRSP)

Cefepime=8 (MSSA, *Streptococcus* spp., *Moraxella*/*H. influenzae*, *E. coli*/*K.pneumoniae*, *Enterobacter*/*Serratia*/*Citrobacter*, *P.aeruginosa*, *A. baumannii*, PRSP)

Ampicillin-sulbactam=8 (MSSA, *Streptococcus* spp., *E.faecalis*, oral anaerobes, *B.fragilis*, *Moraxella*/*H. influenzae*, *E. coli*/*K.pneumoniae*, *A. baumannii*)

Piperacillin-tazobactam=11 (MSSA, *Streptococcus* spp., *E.faecalis*, oral anaerobes, *B.fragilis*, *Moraxella*/*H. influenzae*, *E. coli*/*K.pneumoniae*, *Enterobacter*/*Serratia*/*Citrobacter*, *P.aeruginosa*, *A. baumannii*, ESBL)

Meeropenem=12 (MSSA, *Streptococcus* spp., *E.faecalis*, oral anaerobes, *B.fragilis*, *Moraxella*/*H. influenzae*, *E. coli*/*K.pneumoniae*, *Enterobacter*/*Serratia*/*Citrobacter*, *P.aeruginosa*, *A. baumannii*, ESBL, PRSP)
